# Strength-Endurance Training Reduces the Hamstrings Strength Decline Following Simulated Football Competition in Female Players

**DOI:** 10.3389/fphys.2018.01059

**Published:** 2018-08-24

**Authors:** Anne Delextrat, Jessica Piquet, Martyn J. Matthews, Daniel D. Cohen

**Affiliations:** ^1^Sport and Health Science Department, Oxford Brookes University, Oxford, United Kingdom; ^2^School of Health Sciences, Centre for Health Sciences Research, University of Salford, Salford, United Kingdom; ^3^Faculty of Life Sciences, Universidad de Santander, Bucaramanga, Colombia

**Keywords:** fatigue, strength, endurance, torque, hamstrings, soccer

## Abstract

Hamstring strains are the most common injury in multiple sprint sports, with inadequate eccentric hamstring strength and fatigue identified as important risk factors. Resistance training interventions aimed at reducing injury risk typically focus on the development of maximum strength, while little is known about the impact of training on hamstring fatigue resistance. The present study compared the effects of strength endurance (SE) with a strength intervention (S) on the eccentric hamstring strength decline induced by a simulated soccer match. Twenty-one female soccer players were randomly assigned to a S group (*n* = 10) or a SE group (*n* = 11). Hamstrings and quadriceps isokinetic concentric and eccentric peak torque (PT) were assessed at 120°.s^-1^ and hamstrings-to-quadriceps ratio (HEcc:QCon) calculated, pre- and immediately post a 90-min simulated match (BEAST90). This was repeated following a 7-week intervention of either three to five sets of 6RM leg curl and stiff-leg deadlift with 3-min inter-set rest (S), or the same exercises performed using three sets of 12–20 RM with 45–90 s inter-set rest (SE). At baseline, the simulated match led to significant declines in hamstrings eccentric peak torque (EccPT) in both groups in both dominant (D) and non-dominant (ND) legs [SE: (D: -15.5, ND: -15.6%), *P* = 0.001 to 0.016; S: (D: -12.3%, ND: -15.5%), *P* = 0.001 to 0.018]. After the 7-week intervention, we observed a group^∗^intervention^∗^match interaction such that there was no significant decline in EccPT in the SE group following the simulated match (D: 5.3%, ND: 2.0%), but there remained significant declines in the S group (D: -14.2%, ND: -15.5%, *P* = 0.018–0.001). Similarly, in the SE group, there was a significant decrease in the HEcc:QCon in D before (-14.2%, *P* = 0.007), but not after the training intervention, whereas declines were observed in the S group both at baseline, and following the intervention (D: -13.9%, ND: -15.6%, *P* = 0.045). These results demonstrate that SE training can reduce the magnitude of the EccPT decline observed during soccer competition. As inadequate eccentric strength and fatigue are both risk factors for hamstring injury, SE training should be considered along with the development of peak eccentric strength, as a component of programs aimed at reducing injury risk in multiple-sprint sports.

## Introduction

Resistance training is considered to be an important component of the conditioning practices of soccer teams, both in the context of performance (Silva et al., 2015) and injury risk reduction (McCall et al., 2015a). In soccer and other multiple sprint sports, lower eccentric hamstrings strength assessed in the nordic hamstring exercise (NHE) (Bourne et al., 2015; Opar et al., 2015; Timmins et al., 2016) or using Isokinetic dynamometry (Green et al., 2018) has been identified as a risk factor for hamstrings strain injuries (HSI). There is also weaker evidence that excessive imbalance between the strength of the hamstrings and the quadriceps (hamstrings eccentric to quadriceps concentric strength ratio, H_ecc_:Q_con_) is a risk factor for HSI’s (Croisier et al., 2008; Dauty et al., 2016). Inadequate hamstrings strength may also be risk factors for anterior cruciate ligament (ACL) injuries (Myer et al., 2009; Opar and Serpell, 2014). Resistance training interventions aimed at improving eccentric strength have been successful in reducing HSI incidence in soccer and other sports (Croisier et al., 2008; Goode et al., 2015). As hamstring strains are the most common injuries in soccer (Woods et al., 2004) and other multiple sprint sports (Dick et al., 2007), establishing and implementing an optimal risk reduction conditioning program could have an important impact on player availability.

Despite fatigue also being a well-recognized HSI risk factor (Mendiguchia et al., 2012), only one prospective injury study has evaluated the potential role of poor fatigue resistance (Freckleton et al., 2014). Finding increased in-season HSI incidence in Australian rules footballers who had lower performance in a pre-season posterior chain strength-endurance test. Lord et al.’s (2018) recent finding of significant deficits in isokinetic hamstrings fatigue resistance but not peak torque in the previously injured leg of soccer players with prior HSI, although a retrospective analysis also highlights the need to consider this component of neuromuscular performance in relation to prior injury. Epidemiological evidence suggests that the incidence of both hamstrings and ACL injuries is increased in conditions of greater muscular fatigue, such as in competitions vs. practices, toward the end of the first or second halves vs. earlier in the match (Woods et al., 2004; Waldén et al., 2011). Simulated soccer competition consistently produces large, significant decreases in eccentric hamstrings strength, and as quadriceps peak torque shows little change, there is a progressive increase in the imbalance between these muscle groups across match-play (Greig and Siegler, 2009; Delextrat et al., 2010; Cohen et al., 2015). This data, combined with evidence that biomechanical changes in sprint technique which increase hamstring load parallel hamstring strength declines (Small et al., 2009b), has led to the suggestion that hamstring strength decline may be an important mediator of the higher HSI incidence reported in the latter stages of match play (Greig and Siegler, 2009; Delextrat et al., 2010; Small et al., 2010; Cohen et al., 2015). Potentially, the weak predictive capacity of hamstrings isokinetic peak torque risk screening protocols (Van Dyk et al., 2017), could therefore be partly related to the inherent lack of consideration of the athlete’s capacity to maintain eccentric hamstrings strength and hamstrings:quadriceps strength ratios under the fatiguing conditions during which HSI’s most frequently occur.

Nonetheless, a number of HSI risk reduction conditioning interventions, which have almost exclusively used loading protocols that emphasize the development of maximum strength, have been successful in reducing HSI risk (Goode et al., 2015), supporting the value of this approach. However, it could also be speculated that, at least in players with adequate maximal strength, a greater emphasis on strength-endurance (SE) training and the development of greater hamstring muscle fatigue resistance may be a more effective means to attenuate the decline in hamstrings strength across match-play. In line with this, Matthews et al. (2017) found a significant reduction in hamstring peak torque decline after 45 min of simulated soccer activity following 4 weeks of NHE performed at 12RM (assisted NHE). Similarly, in soccer players, Small et al. (2009a) observed distinct effects on pre-match hamstrings eccentric peak torque (EccPT) and post-match hamstrings EccPT change following a 90-min simulated soccer protocol of a 8-week NHE intervention when the exercise was performed in the warm-up (WU) prior to on-pitch training, compared the cool-down (CD) following it. The CD intervention did not lead to an increase in pre-match hamstrings EccPT, but at half-time (45 min) or full-time (105 min) of the simulated match, EccPT was significantly higher than at the same time points pre-intervention, indicating that training in a fatigued state attenuated strength decline across the duration of simulated match. In contrast, pre-match EccPT significantly increased in the WU group, but showed no improvement at half-time or full-time, indicating that the intervention did not attenuate strength loss during the match. While this approach shows promise as a countermeasure to in-match strength decline, it may not always be convenient to implement resistance training after on-pitch training, and an alternative approach to improve the work capacity of the hamstrings is the development of hamstrings strength-endurance in separate gym settings with conditioning sessions.

Relative to maximum strength (S) orientated training, strength endurance (SE) loading parameters promote larger acute increases in blood lactate (Rogatzki et al., 2014) and greater chronic increases in capillarization and lactate buffering capacity (Campos et al., 2002; Kraemer and Ratamess, 2004), factors which contribute to the increased number of repetitions that can be performed at a submaximal load observed following this type of training (Mitchell et al., 2012). It is not known, however, whether these adaptations or those promoted by maximum strength loading parameters transfer to an improved work capacity and fatigue resistance of the hamstrings during the 90 min of intermittent high-intensity running and other activities that comprise a competitive soccer match.

The aim of the present study was to compare the effects of a high repetition, short inter-set rest period strength-endurance intervention with that of a higher load, longer inter-set rest period strength intervention using the same resistance training exercises, on hamstring strength changes induced by a simulated competitive match in female soccer players. We hypothesized that the strength-endurance intervention would significantly reduce the decline in hamstring EccPT induced by a simulated match match protocol while the strength intervention would not.

## Materials and Methods

### Participants

The study comprised a convenience sample of the 21 female players from the university soccer team, all of whom volunteered to take part in this study. Players were excluded if they were not an outfield player, had a lower leg injury in the past 6 months or a hamstring or ACL injury in the past 2 years. At the time of the study, participants trained for 2 h twice a week, competed in competed in one weekly match at an amateur level (University division 2 and 3 or club county level) and had limited strength training experience as part of the university sports program. Players were recruited via the soccer coach, who explained the study to the whole team and that participation was voluntary.

Players were divided according to playing position and then randomly assigned, by picking names from a hat, to a strength training group, (S group, *n* = 10, age: 21.8 ± 4.0 years; height: 166.2 ± 5.9 cm; body mass: 59.9 ± 9.6 kg, body fat: 21.8 ± 4.9 %, playing experience: 10.0 ± 4.9 years) or a strength-endurance group (SE group, *n* = 11, age: 23.7 ± 7.2 years; height: 165.2 ± 6.9 cm; body mass: 60.5 ± 7.3 kg, body fat: 21.8 ± 3.5 % playing experience: 10.0 ± 3.6 years), such that there was a similar distribution of each playing position in each group. All procedures were in accordance with, and approved by, the University’s Ethical Research Committee standards. Written informed consent to participate in the study was obtained for each participant.

### Procedures

**Table 1** summarizes the timeline of the evaluations and the intervention. Evaluations were performed at baseline and post-intervention (session 1 and session 2). Between baseline and post-intervention, participants performed one of two hamstrings conditioning programs three times weekly for 7 weeks in addition to their soccer training.

**Table 1:** Summary of timeline of evaluations and intervention.

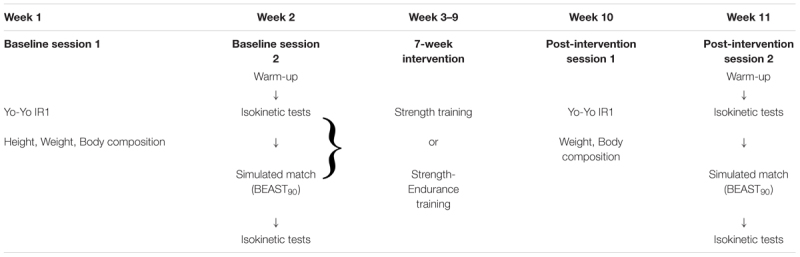

#### Baseline and Post-intervention Assessments

Session 1 consisted of the indirect assessment of maximal oxygen uptake (VO_2max_) using the Yo-Yo intermittent recovery level 1 test (Yo-Yo IR1; Krustrup et al., 2003), as well as anthropometric measurements (at baseline: weight, body composition and height; post-intervention: weight and body composition). This session was conducted during the week prior to session 2, and was included to determine if cardio-respiratory fitness changes had occurred during the intervention which could have an influence on the outcome variables.

Session 2 at baseline and post-intervention, consisted of isokinetic strength tests (Biodex System 2, Shirley, NY, United States), performed immediately before and after performing a simulated soccer protocol (BEAST_90_). The isokinetic tests performed immediately before the BEAST_90_ were preceded by a 10-min warm-up (only in the pre-BEAST_90_ session) on a cycle ergometer (Monark 874E, Varberg, Sweden) with four intermittent 6-s sprints at the 6th, 7th, 8th, and 9th minutes (min). Tests were performed at the same time of the day to minimize performance variations due to circadian rhythms.

During the isokinetic assessments, participants were seated with their hips flexed at approximately 90°. Tests were performed on the dominant (preferred leg used to kick a soccer ball) and non-dominant legs. Stabilization straps to the trunk, thigh, and tibia were attached and the axis of rotation of the dynamometer lever arm was aligned with the lateral femoral condyle. Participants familiarized themselves with each movement by performing six to eight sub-maximal contractions, prior to the five maximal repetitions at a velocity of 120°/s. This velocity was chosen as it has been previously been shown to be highly reliable in the measurement of concentric and eccentric quadriceps and hamstrings peak torque in females (ICCs of 0.82–0.92, Li et al., 1996) and has been used in a number of studies examining interactions between simulated soccer and isokinetic strength (Small et al., 2009a, 2010; Delextrat et al., 2013; Cohen et al., 2015). The range of motion was from 0° (full knee extension) to 90° and players were given encouragement to provide maximal effort throughout the range. One set of quadriceps–hamstrings (extension–flexion) maximal concentric–concentric repetitions was performed, followed by one set of maximal eccentric hamstrings (extension) repetitions with passive return during flexion. From these tests, absolute (N⋅m) peak concentric torque (ConPT) of the quadriceps and hamstrings and peak eccentric torque (EccPT) of the hamstrings were recorded, and further expressed relative to participants’ body mass (%BM). The functional hamstrings-to-quadriceps ratio (H_Ecc_:Q_Con_) was also calculated by dividing hamstrings EccPT by quadriceps ConPT.

The simulated soccer protocol chosen to induce fatigue was the Ball-sport Endurance And Sprint Test [BEAST_90_, (Williams et al., 2010)]. The test consists of repeated circuits during two 45-min halves, separated by 15 min of rest, including sprinting (12-m and 20-m), running at approximately 75% of maximum effort, jogging/decelerating, walking, backward jogging, slaloming between cones, and kicking a football (Williams et al., 2010). Between circuits, participants performed three maximal countermovement jumps (CMJs), starting in an erect position, and performing a downward movement before pushing off from the ground, with hands on their hips throughout. Parameters measured during the test were:

1. 12- and 20-m sprint times (s, timing gates, Brower Timing System, Draper, UT, United States).
2. Circuit times (s, handheld stopwatches, Fastime, Leicestershire, United Kingdom).
3. CMJ height (cm, electronic jump mat, Probotics Inc, United States).
4. Heart rate (HR, beats.min^-1^, Polar V800 heart rate monitors, Warwick, United Kingdom) was continuously measured and expressed as a percentage of the estimated maximal HR (HR_max_) using the Tanaka’s equation (Tanaka et al., 2001).
5. Rate of perceived exertion (RPE) was reported after every circuit (Borg, 1982).

These parameters were further averaged over 15-min bouts (Williams et al., 2010).

Players were familiarized with the protocol by being walked through the course, after the Yo-Yo IR1 in baseline evaluation session 1. The BEAST_90_ was performed outdoors on the same grass pitch at baseline and post-intervention, with a mean temperature 12.8 ± 3.5°C, and humidity of 80.2 ± 4.2%.

The BEAST_90_ has been characterized as a valid and reliable simulation of soccer competition, with key variables such as mean circuit time, sprint times, and heart rate showing a inter-day typical error of measurement of less than 3% (Williams et al., 2010).

To ensure that participants completed the same overall work in the BEAST_90_ at baseline and after the training intervention, as differences could act as a confounding factor when comparing the effects of the training interventions on strength change induced by the protocol, they were encouraged to reproduce their baseline circuit times during the post-intervention BEAST_90_. This was achieved by directing them to slow-down or speed-up before the start of a circuit, if necessary.

#### 7-Week Intervention

Following baseline testing, participants completed a hamstrings conditioning program three times weekly for 7 weeks in a gym, under the supervision of an experienced strength and conditioning coach. Both S and SE groups performed the same exercises, namely the seated hamstrings curl and the stiff-leg deadlift (see **Appendix/Supplementary Materials** for exercises). These exercises were chosen as they are commonly used by recreational athletes and for their complementarity; in the seated hamstrings curl the hamstring muscle group is involved in knee flexion (with hip fixed) while in the stiff-leg deadlift, the muscle group is involved in hip extension (with knee joint fixed) (Schoenfeld et al., 2015). The first session consisted of a five repetition maximum (5RM) test for each exercise, to estimate each participant’s training load (Reynolds et al., 2006).

The seated hamstrings curl exercise was performed on a Cybex Prestige VRS leg curl machine. Participants started in a seated position on the machine with their hips flexed at 90° and their legs extended at 180° on the padded arm of the machine, the lever arm adjusted so that it rested just proximal to the heels. They were then instructed to flex their knees until at least 90° and then to return to the start position in a controlled manner. Stiff-legged deadlifts were performed with dumbbells, arms spaced slightly wider than shoulder width. The exercise started with the dumbbells hanging at arm’s length, trunk extended and scapulae in adduction. Participants were then requested to flex forward at the hips while maintaining a neutral spine (natural lordotic curvature) and knees very slightly flexed, until the back was parallel to the floor or as close to this as comfortably possible, and then return to the start position.

The S and SE groups differed in the intensity (load used), number of repetitions and the length of the inter-set recovery period. The S group performed three to five sets of 6RM with a 3-min inter-set rest, with exercises progressed by increasing both the load and the volume (number of sets). A 5RM test was repeated at the start of the fourth week to adjust the load used. The SE group performed three sets of 12–20 repetitions with 45–90 s inter-set rest period, with progression achieved by reducing the inter-set rest period. See **Appendix/Supplementary Materials** for details of the progression of program variables during the intervention and images of the two exercises used in the study.

### Statistical Analyses

Statistical analyses were performed using SPSS statistical software (version 23.0). The parametric nature of the data was checked using the Shapiro–Wilk test. Subsequently, a three-way ANOVA with repeated measures was used to assess the effects of the match (pre vs. post BEAST_90_), training group (S vs. SE) and timepoint (baseline vs. post-intervention) on the primary outcome measure: hamstrings EccPT, and secondary outcomes: hamstrings ConPT and H_Ecc_:Q_Con_. When significant differences were found, *post hoc* Bonferroni pairwise comparisons were undertaken to identify where they lay. Effect sizes were calculated using Cohen *d* and partial eta squared ($\eta_{p}^{2}$) and interpreted as small (>0.1), medium (>0.3), and large (>0.5). Each dependent variable was presented as mean and standard deviation (Mean ± SD), and 95% confidence interval limits for the differences tested (95% CI) were also shown. We also performed a three-way ANOVA to assess potential interactions between BEAST_90_ performance variables (described above), training intervention and timepoint. For all these analyses, a *P*-value < 0.05 was considered statistically significant.

## Results

### Training Interventions

The loads lifted in the S group were 33.8 ± 3.6 kg and 28.7 ± 3.9 kg, respectively, for the seated hamstrings curl and the stiff-legged deadlift at the start of the intervention 39.2 ± 1.6 kg and 35.2 ± 3.3 kg, respectively, for the same exercises at the end of the intervention. The loads lifted in the SE group were 27.8 ± 6.6 kg and 26.7 ± 3.3 kg, respectively, for the seated hamstrings curl and the stiff-legged deadlift throughout the intervention.

### Effects of Simulated Match on Isokinetic Strength and Interaction With Training Intervention

**Table 2** shows peak torque and *H*_ecc_:*Q*_con_ pre and post BEAST_90_ before and after the 7-week intervention.

**Table 2:** Effect of 7 weeks of strength (S group) and strength-endurance (SE group) training interventions on hamstrings eccentric peak torque (EccPT), hamstrings concentric peak torque (ConPT), and functional hamstrings:quadriceps ratio (H_ecc_:Q_con_) in the dominant (D) and non-dominant (ND) legs of female hockey players.

		Pre-training			Post-training		
			
		Pre-BEAST_90_	Post-BEAST_90_	% change	Pre-BEAST_90_	Post-BEAST_90_	% change
**Hamstrings EccPT (% of body mass)**							
S	D	117.6 ± 34.3	103.1 ± 37.1	-12.3	136.6 ± 36.0	115.4 ± 34.0	-15.5
SE	D	132.9 ± 66.8	112.3 ± 56.4	-15.5	142.6 ± 64.6	135.1 ± 60.4	-5.3
S	ND	113.2 ± 53.0	97.4 ± 43.7	-14.0	130.7 ± 58.2	112.2 ± 48.4	-14.2
SE	ND	128.2 ± 56.7	108.2 ± 52.3	-15.6	136.8 ± 57.1	134.1 ± 57.3	-2.0
**Hamstrings ConPT (% of body mass)**							
S	D	72.8 ± 24.5	66.8 ± 25.2	-8.2	86.8 ± 26.1	79.7 ± 29.3	-8.2
SE	D	83.2 ± 22.5	77.5 ± 25.9	-6.9	92.5 ± 22.1	90.2 ± 20.9	-2.5
S	ND	70.2 ± 19.6	66.0 ± 18.5	-6.0	85.1 ± 19.7	74.7 ± 22.7	-12.2
SE	ND	80.5 ± 17.1	74.0 ± 18.5	-8.1	90.6 ± 23.4	85.6 ± 24.3	-5.5
**H_*ecc*_:Q_*con*_**							
S	D	0.81 ± 0.16	0.73 ± 0.13	-9.9	0.94 ± 0.16	0.83 ± 0.15	-11.7
SE	D	0.82 ± 0.17	0.75 ± 0.17	-8.5	0.85 ± 0.16	0.83 ± 0.15	-2.4
S	ND	0.82 ± 0.22	0.75 ± 0.23	-8.5	0.91 ± 0.20	0.78 ± 0.20	-14.3
SE	ND	0.88 ± 0.16	0.81 ± 0.024	-8.0	0.91 ± 0.14	0.88 ± 0.16	-3.3


The three-way ANOVA revealed significant group^∗^match^∗^intervention interactions which were then followed up.

#### Hamstrings EccPT

**FIGURE 1:**
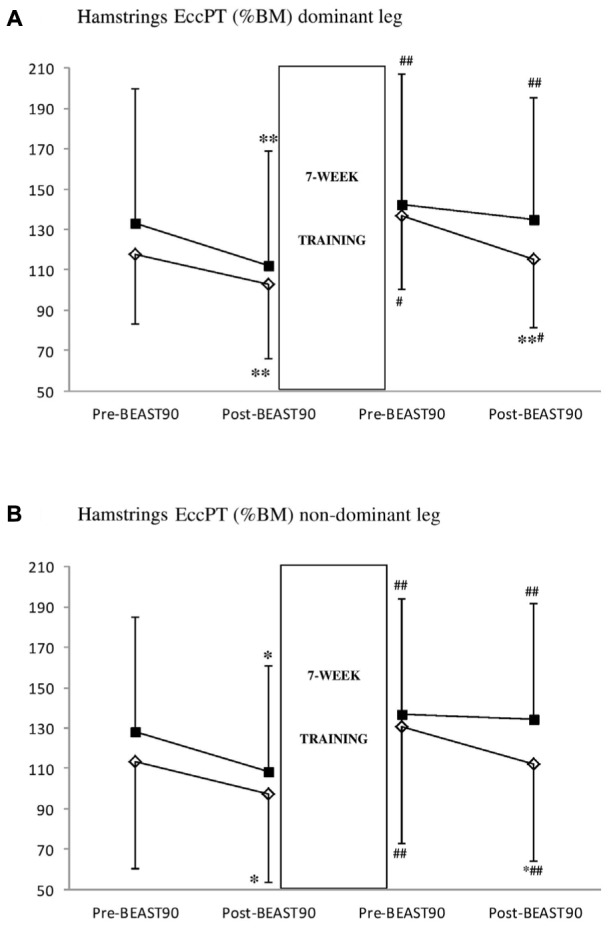
Effect of 7 weeks of strength (S group, empty lozenge symbols) and strength-endurance (E group, full square symbols) training interventions on hamstrings eccentric peak torque [EccPT, % of body mass (BM)] in the dominant **(A)** and non-dominant **(B)** legs. Significantly different from pre-BEAST_90_, ^∗^*P* < 0.05 and ^∗∗^*P* < 0.01. Significantly different from pre-intervention, ^#^*P* < 0.05 and ^##^*P* < 0.01.

**Figure 1** shows that in the SE group, there were significant match^∗^intervention interactions (*P* = 0.017, $\eta_{p}^{2}$: 0.49). At baseline, there was a significant decline in hamstrings EccPT post-match relative to pre-match values in the dominant (*P* = 0.001, *d*: 0.33, 95% CI: -28.7 to -12.4 %BM) and non-dominant leg (*P* = 0.009, *d*: 0.37, 95% CI: -33.6 to -6.3 %BM). After the (SE) training intervention there was no significant post-match decline in EccPT in either leg (*P* = 0.225).

In the S group, there were no significant match^∗^intervention interactions (Dominant: *P* = 0.657, $\eta_{p}^{2}$: 0.04; Non-Dominant = 0.266, $\eta_{p}^{2}$: 0.20). There was a significant decline in hamstrings EccPT post-match relative to pre-match at baseline and also after the 7-week training intervention in the dominant (Baseline: -12.3%, Post-intervention: -15.5%; *P* = 0.001, $\eta_{p}^{2}$: 0.87, 95% CI: -24.8 to -10.9 %BM) and the non-dominant leg (Baseline: -13.9%; Post-intervention: -14.2%; *P* = 0.018, $\eta_{p}^{2}$: 0.70, 95% CI: -29.9 to -4.4 %BM).

#### Hamstrings ConPT and H_Ecc_:Q_Con_

**FIGURE 2:**
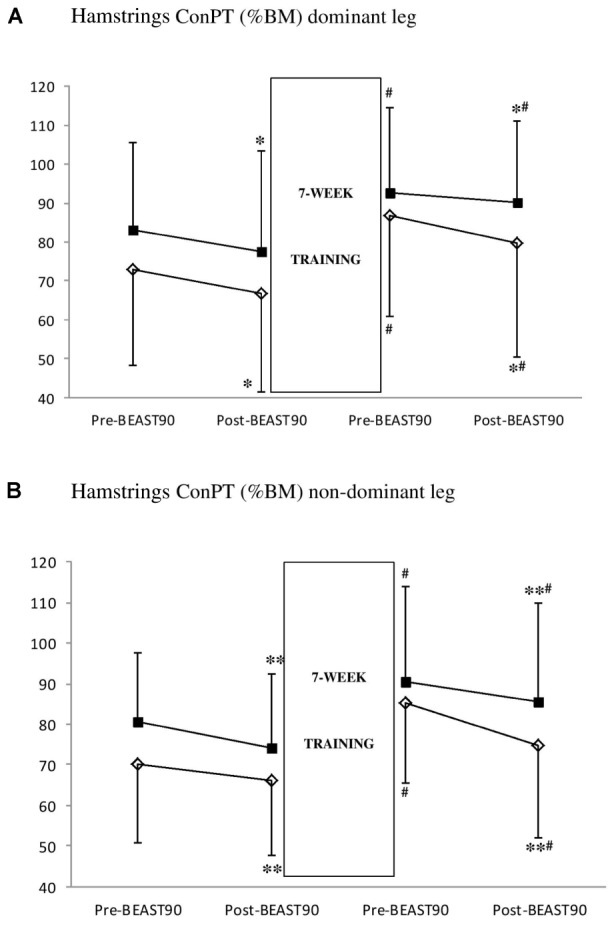
Effect of 7 weeks of strength (S group, empty lozenge symbols) and strength-endurance (E group, full square symbols) training interventions on hamstrings concentric peak torque [ConPT, % of body mass (BM)] in the dominant **(A)** and non-dominant **(B)** legs. Significantly different from pre-BEAST_90_, ^∗^*P* < 0.05 and ^∗∗^*P* < 0.01. Significantly different from pre-intervention, ^#^*P* < 0.01.

**Figure 2** shows that there were no significant match^∗^intervention interactions (*P* = 0.240 and *P* = 0.581, respectively, in the dominant and non-dominant legs) on hamstrings ConPT in either group. Both groups showed a significant decline in hamstrings ConPT post-match relative to pre-match at baseline and also after the 7-week training intervention in the dominant (Baseline: -6.9 to -8.2%, Post-intervention: -2.5 to -8.2%; *P* = 0.025, *d* = 0.11–0.26, 95% CI: -9.9 to -0.75 %BM) and the non-dominant leg (Baseline: -6.0 to -8.1%; Post-intervention: -5.5 to -12.2%; *P* = 0.001, *d* = 0.21–0.49, 95% CI: -9.7 to -3.3 %BM).

**FIGURE 3:**
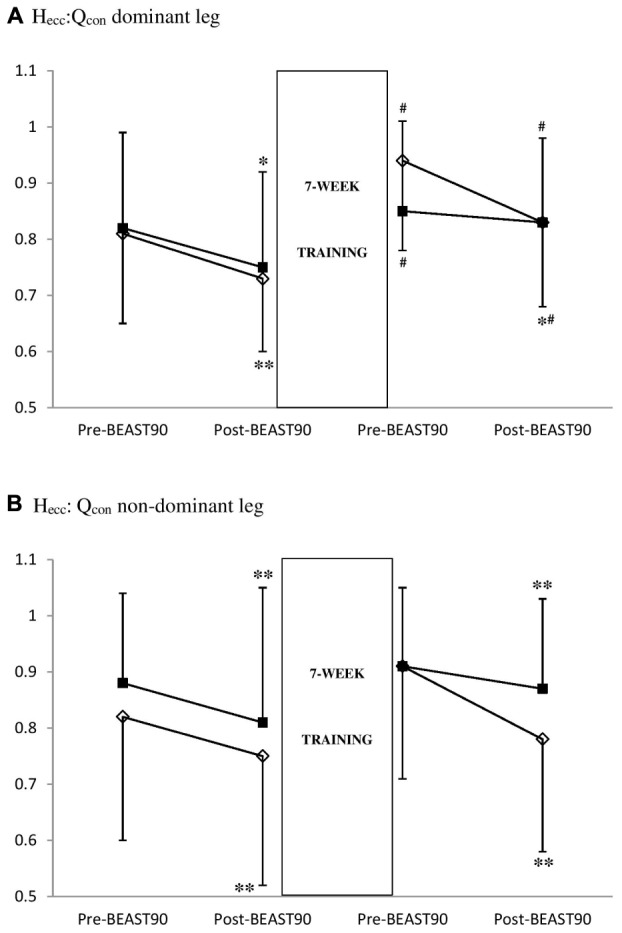
Effect of 7 weeks of strength (S group, empty lozenge symbols) and strength-endurance (E group, full square symbols) training interventions on the eccentric hamstrings: concentric quadriceps ratio (H_ecc_:Q_con_) in the dominant **(A)** and non-dominant **(B)** legs. Significantly different from pre-BEAST_90_, ^∗^*P* < 0.05 and ^∗∗^*P* < 0.01. Significantly different from pre-intervention, ^#^*P* < 0.01.

**Figure 3** shows that there was a significant group^∗^intervention ^∗^match interaction on dominant leg H_Ecc_:Q_Con_ (*P* = 0.039, $\eta_{p}^{2}$: 0.42). In the SE group, there was a significant intervention^∗^match interaction on H_Ecc_:Q_Con_ (*P* = 0.045, $\eta_{p}^{2}$: 0.38), with a significant decrease post-match before (-14.2%, *P* = 0.007, 95% CI: -0.13 to -0.03, *d*: 0.64), but not after the training intervention (*P* = 0.393). In the non-dominant leg, we did not observe any significant intervention effect. In the S group, we observed significant decreases in dominant and non-dominant H_Ecc_:Q_Con_ post-match both at baseline and post-intervention (-13.9% to -15.6%, *P* = 0.045, $\eta_{p}^{2}$: 0.59, 95% CI: -0.19 to -0.01, *d*: 0.33–0.55).

#### Quadriceps ConPT

There was no significant interaction of quadriceps ConPT with any independent variable (*P* > 0.05), except a significant post-match decrease in the dominant leg (-4.1 to -7.9%, *P* = 0.015, $\eta_{p}^{2}$: 0.32). Values ranged from 133.4 %BM to 161.1 %BM in the dominant leg and from 133.8 %BM to 148.9 %BM in the non-dominant leg.

### Time-Dependent Changes in Performance Variables During the Simulated Match, and Interactions With Training Group and Timepoint

**Table 3** shows that performance variable during the match at baseline and post-intervention. At baseline, the simulated match resulted in significant time-dependent decreases in sprint performance (increased 20-m time, *P* = 0.048, $\eta_{p}^{2}$: 0.33) and jump height (15–30 min vs. 75–90 min, *P* = 0.003, $\eta_{p}^{2}$: 0.57), and significant increases in RPE (0–15 min vs. 15–30 min, 30–45 min and 75–90 min, and 45–60 min vs. 30–45 min, *P* = 0.001, $\eta_{p}^{2}$: 0.86) and HR (45–60 min vs. 30–45 min, *P* = 0.045, $\eta_{p}^{2}$: 0.31). There were no interactions between these variables and training group or intervention (*P* > 0.05). This indicates that there were progressive changes in these variables during the simulated match indicative of fatigue, and that neither intervention affected these changes.

**Table 3:** Sprint and vertical jump (VJ) performances, circuit time, ratings of perceived exertion (RPE) and heart rate (HR) during the BEAST_90_ before and after the 7-week training intervention (values are averages of both groups).

	12-m (s)		20-m(s)		Circuit(s)		VJ (cm)		RPE		HR (%HR_max_)	
						
	Baseline	Post-training intervention	Baseline	Post-training intervention	Baseline	Post-training intervention	Baseline	Post-training intervention	Baseline	Post-training intervention	Baseline	Post-training intervention
0–15	2.46 ± 0.20	2.47 ± 0.25	3.78 ± 0.35	3.83 ± 0.60	208 ± 13	207 ± 13	16.2 ± 1.4	15.3 ± 2.2	12 ± 2	11 ± 2	82.1 ± 6.5	80.8 ± 6.7
15–30	2.56 ± 0.30	2.49 ± 0.19	3.93 ± 0.40	4.04 ± 0.44	202 ± 13	194 ± 18	15.7 ± 1.5	15.1 ± 2.5	14 ± 2^∗∗^	13 ± 2^∗∗^	84.5 ± 9.2	82.8 ± 8.6
30–45	2.57 ± 0.29	2.55 ± 0.25	3.93 ± 0.21	4.21 ± 0.55	206 ± 11	202 ± 12	15.7 ± 1.4	15.2 ± 2.5	14 ± 2^∗^	14 ± 2^∗^	85.0 ± 7.8^$^	83.4 ± 8.7^$^
45–60	2.48 ± 0.10	2.58 ± 0.39	4.11 ± 0.43	4.26 ± 0.68	213 ± 7	200 ± 8	15.2 ± 1.1	14.6 ± 2.2	13 ± 2	13 ± 2	82.4 ± 6.4	81.7 ± 8.8
60–75	2.60 ± 0.24	2.66 ± 0.26	4.15 ± 0.44	4.26 ± 0.59	203 ± 11	196 ± 9	15.3 ± 1.3	15.1 ± 2.2	15 ± 2	14 ± 2	83.3 ± 6.8	81.7 ± 8.2
75–90	2.58 ± 0.21	2.63 ± 0.23	3.95 ± 0.25	4.29 ± 0.45	202 ± 9	196 ± 13	14.8 ± 1.2^∗^	15.0 ± 2.3^∗^	15 ± 2^∗$^	15 ± 2^∗$^	85.1 ± 7.0	82.6 ± 8.0


*^∗^Significantly different from 15 to 30 min, *P* < 0.05. ^∗∗^Significantly different from 15 to 30 min, *P* < 0.01. ^$^Significantly different from 45 to 60 min, *P* < 0.05.*

### Effects of Training Intervention on Pre-match Isokinetic Strength

After their respective training interventions both training groups showed significant improvements in all of the pre-match hamstrings isokinetic strength variables, as well as dominant limb Hecc:Qcon. There were no significant changes in quadriceps strength in either group. There were no significant training group^∗^timepoint interactions.

EccPT significantly increased in the dominant (SE: +7.3, *P* = 0.018, CI: +3.5 to +11.8 %BM, *d*: 0.15; S: +16.2%, *P* = 0.001, CI: +10.9 to +24.8 %BM*, d*: 0.54) and non-dominant leg (SE: +6.7%, *P* = 0.020, CI: +2.6 to +15.9 %BM*, d*: 0.15; S: +15.5%, *P* = 0.001, CI: +9.7 to +23.9 %BM*, d*: 0.31). ConPT significantly increased in the dominant (SE: +11.2, *P* = 0.01, CI: +2.8 to +16.9 %BM*, d*: 0.42; S: +19.2%, *P* = 0.002, CI: +8.9 to +28.6 %BM*, d*: 0.55) and non-dominant leg (SE: +12.4, *P* = 0.03, CI: +6.8 to +19.6 %BM*, d*: 0.49; S: +21.2%, *P* = 0.001, CI: +14.3 to +28.3 %BM*, d*: 0.76). H_Ecc_:Q_Con_ significantly increased in the dominant leg (SE: +4.9%, *P* = 0.039, CI: +0.01 to +0.08 %BM, *d*: 0.25; S: +14.6%, *P* = 0.01, CI: +0.05 to +0.22 %BM, *d*: 0.73).

#### Other Variables

There was no significant difference in VO_2max_ post-intervention (42.5 ± 3.6 mL.kg^-1^.min^-1^) relative to baseline (41.3 ± 4.5 mL.kg^-1^.min^-1^).

## Discussion

Studies have consistently reported significant decreases in hamstring EccPT and H_Ecc_:Q_Con_ following simulated football competition (Greig and Siegler, 2009; Delextrat et al., 2010; Small et al., 2010; Cohen et al., 2015). The present study is the first to show that a strength-endurance training intervention can prevent these declines while a strength emphasis intervention using the same exercises had no effect on simulated-match-induced decline in hamstrings strength or H_Ecc_:Q_Con_. This suggests that adaptations associated with strength endurance training may be an effective strategy to protect against hamstrings strength decline during the latter stages of match-play and in turn have an important role in reducing vulnerability to hamstring and ACL injury during the part of the match identified as the highest risk period for injuries (Woods et al., 2004; Waldén et al., 2011).

### Effect of S and SE Training Interventions on Peak Torque and Peak Torque Decline After Simulated Match

The most important finding of the present study was that a 7-week SE training intervention characterized by high repetitions, low-moderate load (12–20 RM) and short inter-set rest periods (45–90 s) significantly reduced the hamstring EccPT decline observed after the simulated soccer match protocol. Prior to the intervention the simulated match led to had large and significant reductions in EccPT in both legs of approximately 15% while only small and non-significant declines were noted after 7 weeks of SE training.

In contrast, the S group who performed the same exercises, but at a higher intensity, with fewer repetitions (6RM), and a longer inter-set rest period still showed a significant match induced decline in hamstrings EccPT. As expected, the non-fatigued state (pre-match) gains in hamstrings peak torque were larger following S; gains of 15–16% in EccPT and 19–21% in Con PT compared to improvements of 6–7% in EccPT and 11–13% in ConPT in the SE group.

### Effect of Simulated Match on Hamstrings and Quadriceps Peak Torque

The baseline match induced decreases in hamstrings EccPT (-12.3 to -15.6%) in both legs, and significant decreases in the H_Ecc_:Q_Con_ (-8.0 to -9.9%) in the dominant leg are comparable to those reported in the literature using other simulated match protocols, such as LIST or SAFT_90_, in male and female soccer players (Rahnama et al., 2003; Small et al., 2009b; Delextrat et al., 2010, 2013; Cohen et al., 2015; Coratella et al., 2017). In contrast, the significant decreases in hamstrings ConPT (-6.0 to -8.2%) and quadriceps ConPT (-5.7 to -7.9%) conflict with most previous studies which found no significant decrease in ConPT in either muscle group after simulated soccer (Greig and Siegler, 2009; Small et al., 2009b; Delextrat et al., 2013; Cohen et al., 2015). This suggests that the BEAST_90_, including 90° changes of direction, and a more varied pattern of movements than other simulated football protocols, may be a more fatiguing protocol, or at least puts a greater demand on force production in the quadriceps and hamstrings. In favor of this hypothesis, Hader et al. (2014) observed that adding 90° changes of directions to a high-intensity intermittent exercise protocol led to fatigue-induced modifications in the control of the lower limb, in particular a reduction in hamstrings activity and a potential mechanical loss in knee stability. However, the relatively low level of conditioning in the present population could also be a contributory factor to the greater fatigue observed. The protective effect of the SE training intervention on the H_Ecc_:Q_Con_ in the dominant leg only could also be due to the characteristics of our simulated football protocol. Indeed, since the effects of both training interventions on hamstrings EccPT did not differ between legs, this variation must relate to differences in quadriceps strength change. Within this context, we observed a post-match decrease in quadriceps ConPT in the dominant leg only, conflicting with previous simulated soccer studies, where no significant decrease was observed in either leg (Greig and Siegler, 2009; Small et al., 2009b; Delextrat et al., 2013; Cohen et al., 2015). These contrasting results could be due to inclusion of the six dominant leg shots after every circuit in the BEAST_90_ protocol, leading to an increase in dominant limb quad fatigue, whereas the LIST or SAFT_90_ used in previous studies comprise running only.

### SE Training Intervention

To our knowledge, only one previous study has compared the effect of two metabolically distinct hamstrings conditioning interventions using the same exercises on work capacity during a simulated or real soccer competition. However, rather than create the challenging metabolic environment by manipulation of the loading parameters of the conditioning exercises as we did, both groups in Small et al. (2009a) performed the same protocol of 6–12 repetitions of the NHE, but one did so in a fatigued state; during the cool-down after an on-pitch training session (CD group), and the other in a non-fatigued state; during the warm-up for the on-pitch session (WU group). In these male players, they found that in the fatigued (CD) group both hamstrings EccPT and H_Ecc_:Q_Con_ were significantly higher post simulated match compared to post match values prior to the 8-week intervention (Hamstrings EccPT: 278.6 ± 37.7 N⋅m vs. 237.9 ± 30.7 N⋅m), but there was no improvement in pre-match values. And similar to our S group, their non-fatigued (WU) group increased fresh state pre-match strength values but post-match values (+17.4 ± 9.7% and -3.5 ± 10.4%, respectively) for pre- and post-match changes were not improved.

While the present study is the first to compare the effects of a strength versus a strength-endurance intervention on the profile of eccentric hamstring strength changes induced by a 90-min simulated soccer protocol, Matthews et al. (2017) evaluated these changes during a 45 min first-half only simulated soccer protocol after 4 weeks of the NHE performed at either 12RM (assisted NHE) or 4RM (unassisted/loaded NHE). They showed that the decline was significantly attenuated by both protocols but the magnitude of change was not significantly different between 12RM and 4RM groups. The lack of between group differences in effect on work capacity, and discrepancy with the present findings may be related both to the lower challenge to fatigue resistance generated by the shorter simulated protocol and to the less distinct loading parameters in their training group. The maximum number of repetitions they performed was 12 and the inter-set rest period was 2 min (the same as used in their strength group), compared to recommendations of 15–25 repetitions and <1-min for muscular endurance (SE) training (American College of Sports Medicine, 2009, stand progression models in resistance training for healthy adults). As we had previously observed that the hamstring EccPT decline following simulated soccer was correlated with blood lactate levels ([La-]_b_, an indirect marker of muscle pH) during the final 15 min of the protocol (Delextrat et al., 2010), our aim was to implement a conditioning protocol which would expose hamstring muscle fibers to high levels of [La-]_b_, speculating that this would drive adaptations that enhance the capacity of those fibers to resist the metabolic fatigue promoting effects of lactate/reduced muscle pH. A number of physiological adaptations to muscular endurance have been identified which may underlie the improvements in hamstring work capacity/fatigue resistance that we observed. Increased capillarization and muscle oxidative capacity have been shown to reduce the production of lactate via decreasing dependence on anaerobic glycolysis, and an improvement in the capacity to buffer lactate/H+ can occur (Campos et al., 2002; Kraemer and Ratamess, 2004; Mitchell et al., 2012) via an increase in the density of muscle cell MCT1 and MCT4 Lactate/H+ transporters and/or activity, enhancing the rate of removal from the muscle fiber where H+ accumulation interferes with excitation and contraction coupling (Juel et al., 2004; Thomas et al., 2012). Our results suggest that our training program promoted local adaptations in the hamstrings, rather than a systemic improvement in endurance, since a significant group^∗^match^∗^intervention interaction were observed on hamstrings EccPT, but not on quadriceps ConPT, or on sprint or jump performance.

Time under tension/number of repetitions and the length of the inter-set rest period are key determinants of the acute [La-]_b_ response to a resistance training protocol (Rogatzki et al., 2014; Weakley et al., 2017). Rogatzki et al. (2014) showed that mean lactate increase from rest to post exercise was significantly higher after an SE protocol consisting of two sets of 20 repetitions at 53% of 1RM with 45 s rest between sets (6.1 mM) than after an S protocol of five sets of 85% 1RM, with 3 min rest (3.9 mM), and higher but not significantly than a hypertrophy protocol consisting of three sets of 70% 1RM with 2 min rest (4.9 mM). Chronic SE training leads to greater improvements in the number of repetitions completed before failure at submaximal loads (such as 30 or 60% of 1-repetition maximum) but lower gains in maximal strength than S training protocols (Campos et al., 2002; Mitchell et al., 2012). Isolated performance tests in which work or repetitions completed at a specific submaximal intensity or workload are typical measures of strength-endurance (Campos et al., 2002; Kraemer and Ratamess, 2004; Mitchell et al., 2012), Spencer et al. (2016) highlight as a proxy measure for work capacity. Work capacity, defined as the ability of the musculature to produce or tolerate variable intensities and durations of work close to the intensity and duration required for sporting performance (Siff, 2003) may therefore be better assessed by comparing muscle performance before and after protocols such as the BEAST_90_ or repeated sprints (Lord et al., 2018) which simulate the activity patterns or key activities of the sport. It has been suggested that the hamstrings are more inherently more susceptible to fatigue than the quadriceps due to a greater proportion of the more fatigable type II muscle fibers (Garrett et al., 1984; Barclay, 1996). However, Sangnier and Tourny-Chollet (2007) argue that the stimulus generated by training and competition must also contribute to this imbalance in fatigue resistance, suggesting that the typical soccer training stimulus may promote inadequate development of hamstring fatigue resistance relative to the development of this quality in the quadriceps.

Epidemiological data shows a time-dependent increase in injury incidence and the highest rates of injury in the final 15 min of competitive match play (Woods et al., 2004; Walden et al., 2012) which temporally aligns with declines in hamstrings EccPT (Greig and Siegler, 2009; Delextrat et al., 2013; Cohen et al., 2015; Coratella et al., 2017) and with changes in sprint technique thought to increase vulnerability to hamstring strain (Small et al., 2010). Against this background, and taking into account that muscle strength and muscle fatigue resistance have been shown to be independent qualities (Jacobs et al., 2005) or inversely related (Bosquet et al., 2015), our findings suggest that the development of hamstrings work capacity via SE protocols, or conditioning in a fatigued state (Small et al., 2010) could be an important component of hamstring injury risk reduction programs in multiple sprint sports, and in athletes with adequate strength might be a more effective approach than increasing maximal strength. Prospective evidence from Aussie rules football shows that poorer muscular endurance, characterized by the number of repetitions of a hamstring/posterior chain bridge exercise completed in a pre-season assessment, was associated with a higher risk of a HSI during the season (Freckleton et al., 2014). Furthermore, Verrall et al. (2005) reported a significant decrease in the number of, and number of players with, hamstring strains following the implementation of a training program which included hamstrings exercises performed in a fatigued state. In addition, as hamstring fatigue is associated with changes in landing biomechanics which increase ACL loading and potentially risk of injury (Ortiz et al., 2010; Thomas et al., 2010), attenuation of hamstrings strength or rate of force development decline across a match could also be beneficial in ACL injury risk reduction.

To our knowledge, the impact of the exercises used in the present intervention – the stiff-leg deadlift and the seated leg curl – on prospective injury risk has not been specifically evaluated. Furthermore, in terms of reduction of HSI incidence, eccentric strengthening of the hamstrings was the most effective approach and the Nordic hamstring, as an isolated intervention has been shown to reduce hamstring injury incidence (Croisier et al., 2008; Goode et al., 2015). While we emphasized control during the eccentric phase of the exercise, in the present study do not specifically overload eccentric strength, and are not considered key components of risk reduction conditioning programs. While this is a limitation of the study, our aim was not to define the response to these specific exercises or to a more realistic mixed conditioning protocol, it was to evaluate the relative effect of metabolically distinct stimui/loading parameters on fatigue resistance rather than define the response to these specific exercises or to a more realistic mixed conditioning protocol. Nonetheless, this approach allowed us to assess the impact of differing loading parameters on adaptations in a relatively short training intervention, and more importantly from a practical perspective, the transfer of strength-endurance focused conditioning to the capacity to maintain eccentric hamstring strength during a 90-min simulated competition. While identifying athlete’s that would benefit from a specific strength endurance orientated intervention by implementing pre and post isokinetic strength testing the BEAST90 or a similar simulated match protocol to is not practical in mostsports settings, there are other means to profile hamstring strength endurance (Freckleton et al., 2014; McCall et al., 2015b; Lord et al., 2018), which should be considered as part of athlete screening.

## Conclusion

The present study confirms a number of previous studies showing a significantly higher decline in hamstrings eccentric than quadriceps concentric peak torque during simulated soccer competition. We found that in female amateur soccer players, a 7-week strength-endurance emphasis hamstrings conditioning program led to a significant reduction in the magnitude of hamstrings EccPT and functional H_Ecc_:Q_Con_ ratio decline induced by simulated competition, while a maximum strength emphasis program using the same exercises did not. Considering these findings in combination with previous experimental evidence (Small et al., 2009b, 2010; Delextrat et al., 2013), as well as epidemiological (Woods et al., 2004) and prospective (Freckleton et al., 2014) and retrospective injury data (Lord et al., 2017), we suggest that “risk screening” and secondary prevention for hamstring, and potentially ACL injuries in multiple-sprint sports should aim to identify athletes with lower levels of both hamstring strength endurance and peak eccentric strength. A training prescription informed by this information and tailored to provide a greater emphasis on S or SE development might enhance the success of risk reduction programs beyond that based on maximum strength development alone. However, further research is needed to evaluate the impact of additional hamstring strength endurance training on hamstrings and ACL injuries. In addition, these findings should be confirmed both in males, and in higher level players of both sexes, as our results may not be generalizable to these populations.

## Author Contributions

AD took part in all the steps: study design, testing, statistical analyses, and manuscript writing. JP took part in testing, statistical analyses, and manuscript writing (Materials and Methods). MM took part in study design and manuscript writing (Introduction and Discussion). DC took part in study design and manuscript writing (all sections).

## Conflict of Interest Statement

DC is a shareholder in a company that develops and supplies force platform hardware and software. The remaining authors declare that the research was conducted in the absence of any commercial or financial relationships that could be construed as a potential conflict of interest.
