# Supplementary material for: Strength-Endurance Training Reduces the Hamstrings Strength Decline Following Simulated Football Competition in Female Players
Source: Front Physiol. 2018 Aug 24;9:1059. doi: 10.3389/fphys.2018.01059 (PMC6138075; doi:10.3389/fphys.2018.01059)
Supplement: Supplementary file 1 [file Data_Sheet_1.docx]

**STRENGTH**
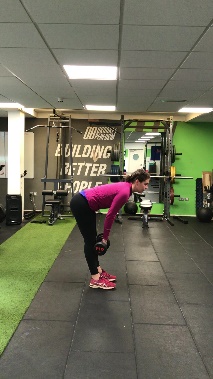

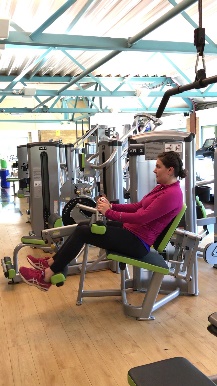


**PROGRAM Straight leg deadlift hamstring curl**

|  | Session 1 | Session 2 | Session 3 |
| --- | --- | --- | --- |
| Week 1 | Test for 6RM: write down the result for hams curl:_________  Dead lift:__________ | 3 x 6 repetitions at 80%of 6RM  3min recovery | 3 x 6 repetitions at 80%of 6RM  3min recovery |
| Week 2 | 3 x 6 repetitions at 90%of 6RM  3min recovery | 3 x 6 repetitions at 90%of 6RM 3min recovery | 3 x 6 repetitions at 90%of 6RM 3min recovery |
| Week 3 | 3 x 6 repetitions at 100%of 6RM  3min recovery | 3 x 6 repetitions at 100%of 6RM  3min recovery | 3 x 6 repetitions at 100%of 6RM  3min recovery |
| Week 4 | Start the session by a “test” of 6RM and write the new load, it will be your new 6RM  hams curl:_________  Dead lift:__________  3 x 6 repetitions at 100%of new6RM  3min recovery | 3 x 6 repetitions at 100%of new6RM 3min recovery | 3 x 6 repetitions at 100%of new6RM 3min recovery |
| Week 5 | 4 x 6 repetitions at 100%of new6RM  3min recovery | 4 x 6 repetitions at new100%of new6RM  3min recovery | 4 x 6 repetitions at new100%of new6RM  3min recovery |
| Week 6 | 5 x 6 repetitions at 100%of new6RM  3min recovery  (if not possible, 4 or 5 reps only) | 5x 6 repetitions at 100%of new6RM  3min recovery | 5x 6 repetitions at 100%of new6RM  3min recovery |
| Week 7 | 3 x 6 repetitions at 100%of new6RM  3min recovery | 3 x 6 repetitions at 100%of new6RM  3min recovery | 3 x 6 repetitions at 100%of new6RM  3min recovery |
