# Supplementary material for: Strength-Endurance Training Reduces the Hamstrings Strength Decline Following Simulated Football Competition in Female Players
Source: Front Physiol. 2018 Aug 24;9:1059. doi: 10.3389/fphys.2018.01059 (PMC6138075; doi:10.3389/fphys.2018.01059)
Supplement: Supplementary file 2 [file Data_Sheet_2.docx]

**STRENGTH -ENDURANCE**
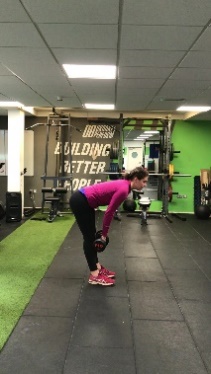

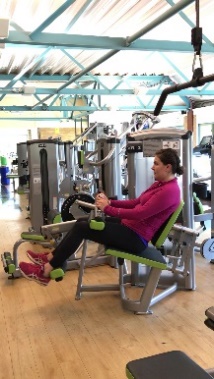


**PROGRAM Straight leg deadlift hamstring curl**

|  | Session 1 | Session 2 | Session 3 |
| --- | --- | --- | --- |
| Week 1 | Test 15RM write down the result for hams curl:_________  Dead lift:__________ | 3 x 12 repetitions at 15RM  1min30 recovery | 3 x 12 repetitions at 15RM  1min30 recovery |
| Week 2 | 3 x 15 repetitions at 15RM  1min30 recovery | 3 x 15 repetitions at 15RM 1min30 recovery | 3 x 15 repetitions at 15RM 1min30 recovery |
| Week 3 | 3 x 18 repetitions at 15RM  1min30 recovery | 3 x 20 repetitions at 15RM 1min30 recovery | 3 x 20 repetitions at 15RM 1min30 recovery |
| Week 4 | 3 x 12-18 repetitions  With only 1min recovery  Write down the number of reps performed and keep the same all week | 3 x 12-18 repetitions  1min recovery | 3 x 12-18 repetitions  1min recovery |
| Week 5 | 3 x 15-20 repetitions  With only 1min recovery  As close as 20 reps as possible, while keeping a short recovery period.  If you can go over 20reps, increase the load. write down the result for hams curl:_________  Dead lift:__________ | 3 x 15-20 repetitions  With only 1min recovery  As close as 20 reps as possible, while keeping a short recovery period.  If possible to increase from 1^st^ session of this week, do so.  If you can go over 20reps, increase the load. | 3 x 15-20 repetitions  With only 1min recovery |
| Week 6 | 3 x 12-18 repetitions  With only 45s recovery  Write down the number of reps performed and keep the same all week | 3 x 12-18 repetitions  45s recovery | 3 x 12-18 repetitions  45s recovery |
| Week 7 | 3 x 12 repetitions  45s recovery | 3 x 12 repetitions  45s recovery | 3 x 12 repetitions  45s recovery |
